# Environment-Aware Rate Adaptation Based on Occasional Request and Robust Adjustment in 802.11 Networks

**DOI:** 10.3390/s23187889

**Published:** 2023-09-14

**Authors:** Weijie Yu, Li Wang, Jin Song, Lijun He, Yanting Wang

**Affiliations:** 1 School of Software, Northwestern Polytechnical University, Xi’an 710072, China; 17710904549@mail.nwpu.edu.cn (W.Y.); 2025180890@mail.nwpu.edu.cn (J.S.); lijunhe@nwpu.edu.cn (L.H.); yantingwang@nwpu.edu.cn (Y.W.); 2 Yangtze River Delta Research Institute, Northwestern Polytechnical University, Taicang 215400, China

**Keywords:** IEEE 802.11, rate adaptation, robustness, NS-3

## Abstract

The IEEE 802.11 standard provides multi-rate support for different versions. As there is no specification on the dynamic strategy to adjust the rate, different rate adaptation algorithms are applied according to different manufacturers. Therefore, it is often hard to interpret the performance discrepancy of various devices. Moreover, the ever-changing channels always challenge the rate adaptation, especially in the scenario with scarce spectrum and low SNR. As a result, it is important to sense the radio environment cognitively and reduce the unnecessary oscillation of the transmission rate. In this paper, we propose an environment-aware robust (EAR) algorithm. This algorithm employs an occasional small packet, designs a rate scheme adaptive to the environment, and enhances the robustness. We verify the throughput of EAR using network simulator NS-3 in terms of station number, motion speed and node distance. We also compare the proposed algorithm with three benchmark methods: AARF, RBAR and CHARM. Simulation results demonstrate that EAR outperforms other algorithms in several wireless environments, greatly improving the system robustness and throughput.

## 1. Introduction

### 1.1. Background

The devices in a wireless local area network transmit and receive data through wireless electromagnetic waves, where the spectrum is extremely limited. This determines that the devices are required to enhance performance cognitively by interaction with the radio environment. In 802.11 networks, the wireless medium is shared among multiple nodes, and channel conditions can be unpredictable due to factors like node mobility, channel fading and interference [1]. As a result, the quality of wireless links can vary over time, leading to potential disruptions in network performance. Therefore, it is crucial for network devices to actively engage with the environment in order to mitigate these influential factors. Although higher rates can result in higher throughout and lower the channel occupancy time, the difficulty of parsing at the receiving end is greater, and messages are not easily received correctly [2]; on the contrary, although lower rates are easier to parse, increasing transmission time affects efficiency and increases the probability of interference. Interference-causing reception errors may result in packet retransmission or even discarding, significantly reducing data transmission efficiency. Any transmission rate that is too high or too low can potentially affect transmission efficiency. Rate selection plays a pivotal role in the implementation of IEEE 802.11 standard. A well-designed rate adaptation algorithm can not only improve data transmission efficiency, but also balance the stability of data transmission [3]. On the contrary, using inappropriate rate adaptation algorithm can ultimately lead to poor internet experience and even inability to communicate properly. Therefore, adaptively selecting an appropriate transmission rate for the given channel quality becomes an important task in improving the performance of each wireless link. This is the task of rate control at the MAC layer.

In the original IEEE 802.11 standard, all frames were transmitted at inherent rates. Due to the continuous changes in channel environment, it is not possible to switch to higher rates to fully utilize channel resources when the channel conditions improve; when channel conditions deteriorate, excessively high rates result in the continuous retransmission of data packets [4]. From this perspective, a single transmission rate will seriously affect the utilization of channel resources and network performance, resulting in a significant reduction in system throughput.

In the subsequent IEEE 802.11 standard, support for different rates was provided by changing the modulation and encoding methods of the physical layer. For example, in 802.11b, four transmission rates are provided: 1 Mbps, 2 Mbps, 5.5 Mbps and 11 Mbps. In 802.11a, eight transmission rates are provided: 6 Mbps, 9 Mbps, 12 Mbps, 18 Mbps, 24 Mbps, 36 Mbps, 48 Mbps and 54 Mbps. Although the IEEE 802.11 physical layer provides support for multiple rates, the standard only specifies the allowed rate set and does not design relevant schemes to achieve rate switching. Because rate selection is crucial for improving the performance of wireless network, the research on this topic has great practical significance for engineering guidance.

In Section 3, we propose an environment-aware robust (EAR) algorithm based on the analysis of various rate adaptation algorithms. The four key contributions of this paper are as follows:

1. EAR uses the method of comparing the packet loss rates of neighboring windows to perceive changes in the channel environment and adjusts the transmission rate accordingly;
2. By using the frequency-based RTS/CTS mechanism, it effectively distinguishes the cause of packet loss whether it is due to low SNR or a hidden terminal;
3. The dynamic window and threshold adjustment mechanism make the real-time and accuracy of rate adjustment much higher than some classic rate adaptation algorithms currently available;
4. The algorithm’s oscillation detection mechanism can effectively suppress or utilize abnormal rate fluctuations, further improving system throughput.

### 1.2. Starting Point

One of the important factors affecting wireless network performance is link layer retransmission. According to the IEEE 802.11 standard, all unicast frames sent require ACK frames for confirmation. If there is a conflict or loss during data transmission, the CRC (Cyclic Redundancy Check) at the receiving end will fail, making it impossible to reply to the sending end with an ACK frame. The transmitter did not receive the ACK frame corresponding to the packet within the SIFS (Short Interframe Space), and will attempt to resend the data frame. In addition, even if the receiving end replies to the ACK frame normally, encountering a loss of the ACK frame can lead to the occurrence of retransmission.

Retransmission will have two levels of impact on the performance of a wireless network. From a micro level perspective, retransmission will increase the overhead of the data link, leading to worsening channel congestion and thereby reducing the throughput of the entire wireless network coverage range of the site. From a macro perspective, for upper layer applications, retransmission can cause message jitter and delay. Real-time application services, such as voice and video calls, heavily rely on continuous message transmission. For these applications, real-time performance is higher than data accuracy. Because even if a short segment is missing from the sound or image, users may not have a clear perception, but due to the high delay caused by retransmission, the discontinuity or lag of the sound or image will greatly affect the normal operation of the application.

There are many explanations as to why retransmission occurs, such as multipath effects [5], radio frequency interference, low SNR, hidden terminal, near/far effects [6], power mismatch and adjacent-channel interference [7], which can all lead to retransmission. Although the multipath phenomenon is a serious problem in traditional 802.11a/b/g devices, with the emergence of 802.11n technology and the application of MIMO (Multiple-Input Multiple-Output) and MRC (Maximum-Ratio Combining) [8] signal processing technology, multipath phenomenon has actually had a positive impact on system throughput. The method of using CSMA/CA media access can effectively avoid near/far effects. In addition, issues such as radio frequency interference, power mismatch, and adjacent frequency interference are usually difficult to solve at the software level. Therefore, perceivable and controllable factors, such as SNR and hidden terminal, have become the key to planning most of rate adaptation algorithms.

In the presence of a hidden terminal, the channel may experience significant bit error and packet loss [9]. The traditional algorithm believes that as long as the bit error and packet loss rate increases, the sender needs to reduce the transmission rate. Actually, doing so does not solve the collision problem [10]. Reducing the transmission rate can lead to more intense channel conflicts due to the prolonged transmission time of each packet, thereby exacerbating the conflicts. This reduction can also cause a further decrease in transmit rate, which could have a detrimental effect on the system’s throughput performance, creating a vicious cycle. Consequently, EAR’s key challenge is to identify the cause of packet loss, whether it is collision or channel noise, and adopt adaptive strategies to regulate the transmission rate in varying environments. Therefore, it is imperative that the EAR be designed in accordance with three fundamental principles:

1. When the wireless network environment is favorable, try to utilize high-rate to transmit data.
2. In situations where the SNR is low, the likelihood of successfully receiving data packets decreases as the transmission rate increases. Therefore, opting for a lower transmission rate becomes imperative.
3. In collision scenarios caused by a hidden terminal, a decrease in transmission rate may actually result in channel deterioration and a subsequent reduction in overall system throughput. As a result, it is imperative to select a more suitable transmission rate.

The remainder of this paper is as follows. Section 2 introduces some research achievements in rate adaptation proposed in recent years. Section 3 explains our proposed EAR scheme. Section 4 explains the configuration of the simulation and its results. Finally, the conclusion is given in Section 5.

## 2. Related Works

In recent years, rate adaptation algorithms have been widely studied and applied in the field of wireless communication. As shown in Table 1, based on different dimensions, the most common classification methods are as follows: based on the sending end or based on the receiving end; and based on historical statistics or based on current measurements [11]. The above classification methods are not completely independent, and in practical applications, it is often necessary to cross the two or combine other classification methods to design appropriate rate adaptation algorithms.

The algorithm based on the sender determines the rate of transmission based on the conditions of the sender itself [22]. The receiver-based algorithm will feedback the appropriate rate to the sender by evaluating the current channel conditions, and adjust the next transmission rate after the sender receives the feedback information [23]. The algorithms based on the sending end include ARF, AARF, ONOE, etc. These algorithms have strong compatibility with various wireless network products and do not need to modify the existing IEEE 802.11 frame format, making them easy to implement. Rate adaptation algorithms based on the receiving end include OAR, RBAR, RARA, and so on. Although these algorithms can theoretically obtain more accurate reference data, additional feedback mechanisms are needed to transmit this information to the sender [24]. Usually, CTS frames or ACK frames are used to transmit feedback information, and it is generally necessary to modify the IEEE 802.11 standard frame structure. Therefore, many algorithms cannot be implemented in existing IEEE 802.11 devices and can only be used for theoretical research.

According to algorithms based on historical statistics or current measurements, channel information can be obtained in principle, but the difference between the two lies in the real-time nature of the information. Historical statistics algorithm using the data transmission situation in the previous period to make predictions about the transmission situation in the next period, thereby making rate adjustment decisions in advance. Therefore, it is generally suitable for environments with slow channel changes, and its performance is not very good for scenarios with drastic changes in channel environment. Typical algorithms based on historical statistics include ARF and the improved AARF algorithm based on ARF. These two algorithms record the success and failure status of each transmission frame to determine whether to adjust the rate.

ARF takes data packets as the statistical unit and starts a timer at the beginning of the algorithm. When the data packet fails to be sent twice in a row, the sender will directly reduce the transmit rate. Whenever 10 consecutive packets are successfully sent or the timer reaches the specified time, a higher rate packet will be sent for detection. If the detection packet is successfully sent, that is, the sending end successfully receives the ACK from the receiving end, then it will be sent at this detection rate next time. If the detection frame fails to be sent, the transmission will continue at the previous rate thereafter. After each rate adjustment, the timer will be reset. To stabilize the rate and reduce the occurrence of rate oscillation, when AARF detects a detection packet transmission failure, it will increase the threshold for the rate increase to twice the original value. For example, if the initial threshold is 10 and the detection packet fails to transmit, the threshold will be updated to 20 (the maximum value does not exceed 50), which means that the next time we want to increase the rate again, we need to successfully transmit 20 consecutive times. If the rate decreases due to two consecutive transmission failures during normal transmission, the threshold for the rate increase will be reset to the initial value of 10. AARF increases the time for rate improvement in a stable channel environment, resulting in lower-rate oscillation. However, the relatively fixed adjustment modes of these two algorithms are difficult to adapt to flexible and ever-changing channel environments. Overall, algorithms based on historical statistics infer the next channel state based on channel states in the past.

In channel-measurement-based methods, it can be further divided into measurement RSS (Received Signal Strength), SNR (Signal-to-Noise Ratio), BER (Bit Error Rate), PLR (Packet Loss Rate) and other indicators [25]. For example, in algorithms such as CHARM and SGRA, the sender determines the channel status by the signal strength RSS value in ACK. CHARM utilizes trial and error to find better transmission rates, and therefore, this mechanism is not well suited for mobile high dynamic environments. Before sending the data packet to a specific destination, CHARM calls the path-loss prediction algorithm to estimate the current path loss to the destination. The sender uses its own transmission power and the noise level at the receiver to obtain the SINR estimation of the receiver. Finally, a set of transmission rates is determined by looking up the SINR threshold table. CHARM utilizes channel reciprocity to enable the sender to obtain relevant receiver information, without incurring RTS/CTS overhead during the algorithm process. Although the combination of these technologies enables CHARM to quickly respond to changes in dynamic channels, CHARM inevitably encounters three problems: (1) The transmission of power and noise level information relies on introducing an additional 802.11 information element into the beacon, probe request and probe response, which violates the existing 802.11 frame format standards. (2) If the sending end is using a power control algorithm, it will have a certain degree of impact on the accuracy of CHARM, depending on the speed of power change. (3) CHARM assumes a symmetric link between the sender and receiver, which is not true in wireless channels due to mobility, fading and interference [26].

In RBAR, the format of the RTS frame is modified to no longer carry the reserved channel time, but rather the rate to be used and the size of the data frame. Neighbor nodes calculate the reserved channel time based on these two values. After receiving the RTS frame, the receiving end selects an appropriate rate based on the SNR value of the RTS frame and carries the rate in CTS to inform the sender. The receiving end defines two SNR thresholds for each rate. If the measured SNR falls precisely between these two thresholds, the rate is selected. The disadvantage of RBAR is that it requires enabling the RTS/CTS mechanism for a prolonged period of time to assist in rate control, which is relatively expensive. In addition, RBAR requires modifications to the frame format of RTS/CTS, which brings compatibility issues. The rapid changes in channel conditions require the responsive adaptation of transmission rates. Ideally, RBAR selects an optimal rate for every frame transmission. However, it requires low-latency hardware that may be dedicated to processing MAC-layer frames, such as FPGA, that is capable of selecting the rate on a per-frame basis. From this perspective, RBAR is also difficult to apply to general network devices.

## 3. Framework and Design

### 3.1. Initialization

In the 802.11 network, each node possesses cognitive abilities to enhance its interaction with the environment. During algorithm initialization, it is necessary to configure several fundamental parameters. Table 2 below provides a list of these parameters along with their respective setting values.

The initial rate $R_{org}$ is set to the highest supported rate under the current protocol, for example, the initial rate of 802.11b is 11 Mbps, and the initial rate of 802.11a/g is 54 Mbps. The window value is the number of statistical packets, which is the foundation for calculating the packet loss rate. The default value of the initial window is set at 30. In addition, during the algorithmic operation, the window size is subject to dynamic updates based on the current packet loss rate, threshold range and rate. The setting of the window range is particularly important for the algorithm. On the one hand, it can prevent the lag of environment perception caused by a window that is too large, and on the other hand, it can reduce the impact of computing overhead and accidental packet loss caused by a window that is too small. In order to ensure that the window size changes within a reasonable range, we combine the above related papers based on window algorithm and our own simulation experience. When the maximum and minimum values of the window are set to 40 and 20, respectively, the algorithm performs well.

After extensive experiments, we have made the following limitations on the range of threshold values: the increase rate threshold range is set at 0.1–0.3; The decrease rate threshold range is set at 0.3–0.5. Initialize $P_{up\_org}$ in the table is the initialization threshold for increasing the rate, which defaults to 0.2. $P_{down\_org}$ is the initialization threshold for reducing the rate, which defaults to 0.4. Set coefficient α to 0.2. It has been demonstrated through simulation that the range of threshold values and the coefficients for updating them can enable the algorithm to perform optimally under this setting.

### 3.2. Packet Loss Analysis

The sending end will compare the number of ACKs received with the total number of packets sent to determine the packet loss rate of this window, The calculation formula for loss is as follows Equation (1).

(1) $$P_{loss}=\frac{Transmitted\_frames-ACK\_frames}{Transmitted\_frames}$$

Among them, Transmitted_frames represents the total number of packets sent during the window period. ACK_frames represents the number of ACK frames received, and subtracting the two represents the number of packet losses during this window. This is in accordance with $P_{loss}$, $P_{up}$ and $P_{down}$, which are used to determine the update strategy for the next window.

### 3.3. Window Update

The setting of window size will greatly affect the performance of the algorithm in different environments. If the window size setting is too small, the packet loss rate statistics will be too contingent, which will result in the wrong rate selection; on the contrary, if the window set is too large, the algorithm will react slowly and cannot adapt to changes in the channel in a timely manner.

Different window sizes can affect transmission times for multiple rates. In high-speed states, increasing the window size results in more data packets being transmitted during the window period. This adjustment is ideal when subsequent channel conditions are well predicted and facilitates the transmission of high-speed data packets. However, to prevent rate increases from causing mismatches with channel conditions, reducing the window size enables timely callback at high rates, avoiding higher packet loss rates. In low-speed states, reducing the window size results in fewer transmission opportunities at that rate, allowing the system to adjust to high-speed rates faster. Nonetheless, when the SNR is too low, the packet loss rate at the lowest rate remains too high. To mitigate this issue, increasing the window size helps to reduce the use of the RTS mechanism and further minimizes system losses. Therefore, EAR proposes an idea of dynamically adjusting the window size, which adopts the following Algorithm 1 based on the current packet loss rate and rate:
**Algorithm 1:** Window Update Algorithm1:  if (Current window is RTS window || Next window is RTS window) { 2:        ${Wind}_{next}={Wind}_{curr}$; 3:        break; 4:   } 5:  else { 6:        $if\;(P_{loss}\leq P_{up})\;\{$ 7:          $if\;\left(R_{curr}<R_{max}\right)\;\{$ 8:              ${Wind}_{next}={Wind}_{min};$ 9:              } 10:         else { 11:             ${Wind}_{next}={Wind}_{curr}+ceil({Wind}_{curr}*(P_{up}-P_{loss}))$; 12:             $if\;\left({Wind}_{next}\geq {Wind}_{max}\right)\;\{$ 13:                 ${Wind}_{next}={Wind}_{max}$; 14:              } 15:         } 16:          } 17:       $else\;if\left(P_{loss}\geq P_{down}\right)\;\{$ 18:              $if\;\left(R_{curr}>R_{min}\right)\;\{$ 19:             ${Wind}_{next}={Wind}_{curr}-ceil({Wind}_{curr}*\left(P_{loss}-P_{down}\right))$; 20:             $if\;\left({Wind}_{next}\leq {Wind}_{min}\right)\;\{$ 21:                  ${Wind}_{next}={Wind}_{min}$; 22:              } 23:             } 24:            else { 25:                ${Wind}_{next}={Wind}_{max}$; 26:             } 27:           } 28:        else { 29:         ${Wind}_{next}={Wind}_{curr}$; 30:         } 31:  }

- When the current or next window is an RTS window, the size of the next window equals the size of the current window.
- When the packet loss rate $P_{loss}$ is less than or equal to $P_{up}$, trigger the rate increase strategy (binary search), and if the rate at this point does not reach the maximum rate supported by the protocol, the size of the next window should be set to the minimum window value defined at initialization. Because if the increased rate is not suitable for the current channel environment, the algorithm can quickly callback the rate and stop the loss as soon as possible when the rate mismatch leads to continuous packet loss.
- Unlike the above situation, the current rate has reached the maximum rate supported by the protocol, and the packet loss rate is low. In order to maximize system throughput, the algorithm allows the high-speed rate to have more transmission opportunities, and increases the window size while maintaining the maximum rate in the next window.
- When the packet loss rate $P_{loss}$ is more than or equal to $P_{down}$, and the rate of the current window is not the lowest rate, the size of next window needs to be reduced. In order to speed up the window size update frequency and increase the probability of sending high-speed data packets. Similar to the logic of window enlargement, it guides the setting of subsequent window sizes based on the current window size and packet loss rate.
- Undertake the above content, and if the current window is the lowest rate, the next window size should be adjusted to the maximum to minimize the use of RTS and reduce resource consumption.
- The threshold of window packet loss rate is between $P_{up}$ and $P_{down}$, and the size of the next window remains unchanged.

### 3.4. Threshold Update

**Figure 1:**
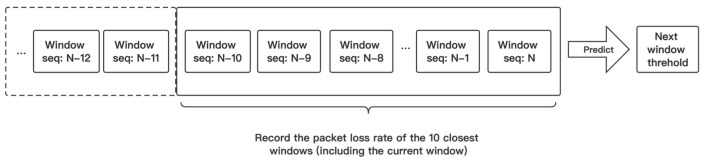
The packet loss rate information of the last ten windows is recorded to calculate the threshold for the following windows.

In addition to elastically scaling the window size according to the current channel environment, the threshold of the window also needs to be adjusted in real time. The dynamic threshold can effectively predict the channel environment, and the rate within the most suitable range can be stabilized. The lower the threshold for increasing the rate, the greater the difficulty in increasing the rate; on the contrary, the higher the threshold for increasing the rate, the simpler it is to increase the rate. The same principle applies to reducing the threshold of the rate. The setting of the threshold needs to be determined before the window executes the sending task. This paper adopts a method similar to EWMA (Exponentially Weighted Moving Average) [27], which guides the subsequent threshold setting through short-term historical thresholds. The threshold update is shown in Figure 1 below.

Inferring the packet loss rate W for the next window based on the recent performance of the packet loss rate, we obtain Equations (2) and (3).

(2) $$W=\alpha *\frac{\sum_{i=1}^{N-1}P_{i}}{N-1}+\left(1-\alpha \right)*P_{N},\;\;\;\;\;\;\;\;\;(1<N<10)$$

(3) $$W=\alpha *\frac{\sum_{i=N-9}^{N-1}P_{i}}{N-1}+\left(1-\alpha \right)*P_{N},\;\;\;\;\;\;(N\geq 10)$$

where $P_{i}$ is the packet loss rate of the N−i history window closest to the current window, and $W\leq \left(P_{up\_org}+P_{down\_org}\right)/2$ and $P_{N}\leq P_{up\_N}$ indicate that packet loss rate is relatively low in the near future, and the next window has met the requirements for increase rate. $P_{N}$ and $P_{up\_N}$ represent the packet loss rate and increase rate threshold of the current window. The $P_{up}$ increase in the next window makes it easier to trigger the mechanism of rate increase to achieve higher throughput, where $W>\left(P_{up\_org}+P_{down\_org}\right)/2$ and $P_{N}\geq P_{down\_N}$ indicates that after inference, not only the current window, but also the next window may have a higher packet loss rate. Then, $P_{down}$ will be decrease to prevent the sustained occurrence of excessive packet loss rate. The pseudocode for threshold update is shown in Algorithm 2 below.
**Algorithm 2:** Threshold Update Algorithm1:  if (Current window is RTS window || Next window is RTS window){ 2:       $P_{up\_next}=P_{up\_curr};$ 3:       $P_{down\_next}=P_{down\_curr};$ 4:   } 5:  else { 6:    $W=0.2\;*\;(sum(history\_loss)/history\_loss.length)+0.8\;*\;P_{loss};$ 7:    $Avg=\left(P_{up\_org}+P_{down\_org}\;\right)/2;\;$ 8:    $if\;(W\leq Avg\;\&\&\;P_{loss}<P_{up\_curr})\;\{$ 9:            $P_{up\_next}=\left(1-W\right)*P_{up\_org}+P_{up\_curr};$ 10:          $P_{down\_next}=$$P_{down\_curr}+W*P_{down\_org};$ 11:          $if\;(P_{up\_next}>0.3)\;\;\{$$P_{up\_next}=0.3;\;\}$ 12:          $if\;(P_{down\_next}>0.5)\;\;\{\;P_{down\_next}=0.5;\;\}$ 13:          break; 14:    } 15:   $if\;(W>Avg\;\&\&\;P_{loss}>P_{down\_curr})\;\{$ 16:          $P_{down\_next}=P_{down\_curr}-W*P_{down\_org};$ 17:          $P_{up\_next}=P_{up\_curr}-\left(1-W\right)*P_{up\_org};$ 18:          $if\;(P_{down\_next}<0.3)\;\{$$P_{down\_next}=0.3;\;\}$ 19:          $if\;(P_{up\_next}<0.1)\;\;\{\;P_{up\_next}=0.1;\;\}$ 20:          break; 21:       } 22:      $P_{up\_next}=P_{up\_curr}$; 23:      $P_{down\_next}=P_{down\_curr};$ 24:   } 25:  $history\_loss.append\left(P_{loss}\right);$ 26:  $if\;\left(history\_loss.length\geq 10\right)\;\{$ 27:   $history.pop\left(0\right);$ 28:  }

### 3.5. Environment-Aware Robust (EAR) Algorithm

After the counter is cleared, calculate the packet loss rate $P_{loss}$ of the current window, and if $P_{loss}\leq P_{up}$, then the sending rate of the next window will increase. In order to find the most suitable rate faster, a binary search method is used to select the rate of the next window; If $P_{down}>P_{loss}>P_{up}$, then the rate within the next window remain unchanged; If $P_{loss}\geq P_{down}$, the rate will not immediately decrease. Instead, add a window ${Wind}_{rts}$ to enable the RTS mechanism later, during this window period, before sending each data packet, an RTS frame is sent to make a reservation for the channel. According to the packet loss rate $P_{rts\_loss}$ during the ${Wind}_{rts}$ period, the following four strategies will be adopted to update the rate:

- If $P_{rts\_loss}\geq P_{loss}$: The packet loss rate not only does not decrease, but also worsens the channel conditions and increases the packet loss rate. This indicates that the reason for the high packet loss rate in the current environment is due to the low SNR. Therefore, the next window should decrease the rate (stepped type) and turn off the RTS mechanism.
- If $P_{down}<P_{rts\_loss}<P_{loss}$: Although the packet loss rate has slightly improved, it still does not fall below the threshold of decrease the rate. In this case, the benefits of enabling RTS are not enough to offset the costs of RTS. Therefore, the next window should maintain the original rate and turn off the RTS mechanism.
- If $P_{up}<P_{rts\_loss}\leq P_{down}$: The packet loss rate has reduced to a certain extent, and the throughput benefits brought by RTS are slightly greater than the inherent costs brought by RTS. The factors leading to a higher packet loss rate in the upper window are mostly caused by hidden station issues. Therefore, the next window maintains the original rate of transmission, and the RTS mechanism is still enabled in the next window.
- If $P_{rts\_loss}\leq P_{up}$: After enabling the RTS mechanism, the packet loss rate has been greatly reduced, indicating that the previous high packet loss rate was entirely caused by hidden terminal issues. At this point, the opening of RTS brings great benefits and eliminates the impact of SNR factors. Therefore, in order to maximize the benefits, the next window increases the rate of transmission and still open RTS mechanism.

**Figure 2:**
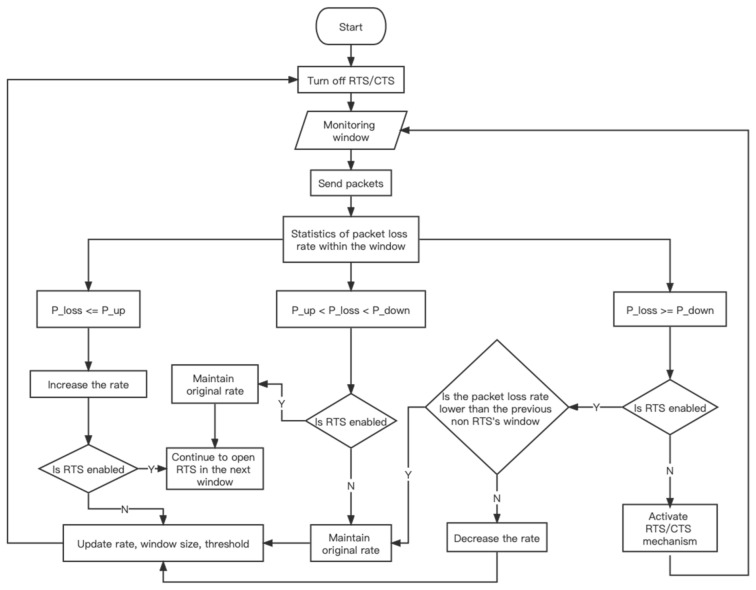
EAR execution flowchart.

In the case of hidden terminal, reducing the rate will make the conflict more intense, as it prolongs the transmission time of each data packet, exacerbating channel conflicts and affecting system throughput. The execution flowchart of the EAR algorithm is shown in Figure 2 below.

### 3.6. Rate Oscillation Detection

In a relatively stable channel environment, if the threshold selection is not appropriate, it may cause the rate selection mechanism to continuously alternate between adjacent high-speed rates, thereby forming rate jitter, which may affect the network throughput [28]. For example, the current speed is 12 Mbps, and the window packet loss rate is 10%, which meets the requirement of increasing the speed to 18 Mbps. Afterwards, the window packet loss rate increased to 50%, and due to the high packet loss rate, it returned to 12 Mbps, repeating this cycle. In theory, if the channel is stable, the corresponding packet loss rate at each rate should be very stable. Therefore, according to calculations, the throughput at 12 Mbps is actually higher than the throughput provided by 18 Mbps. In order to avoid such problems, we added an oscillation detection mechanism to the algorithm: let R be the current rate and $R^{+}$ be the higher-order rate. If the history of rate selection shows a sequence like $R\to R^{+}\to R\to R^{+}\to R\to R^{+}$, it is determined that there is an oscillation reaction at that rate.

When oscillation occurs, in order to maximize throughput, it is necessary to combine the oscillation sequence and compare the average throughput of $R^{+}$ and R. If the average throughput provided by $R^{+}$ is higher than R, the sequence can be ignored and subsequent oscillations can be continuously detected until the oscillation sequence is broken or R’s average throughput is higher than $R^{+}$. If the average throughput provided by R is higher than $R^{+}$, it indicates that the threshold for the rise rate is not set properly. In order to stabilize the current rate, it is necessary to lower the threshold for the rise rate, making the requirements for the rise more stringent.

In order to implement the rate oscillation mechanism, the algorithm needs to continuously record the relevant information of the six windows closest to the current window during execution. Set the window rate sequence as $R_{ol}\;[R_{1},R_{2}\ldots \;R_{6}]$, the window start timestamp sequence as $T_{begin}\;[T_{b_{1}},T_{b2}\ldots T_{b6}]$, the corresponding window end timestamp sequence as $T_{end}\;[T_{e1},T_{e2}\ldots T_{e6}]$, and the number of packets successfully sent by the window sequence as $S_{suc}\;[S_{1},S_{2}\ldots S_{6}]$. If $R_{ol}$ satisfies the law of $R\to R^{+}\to R\to R^{+}\to R\to R^{+}$, then the following Equations (4) and (5) needs to be used to make a detection.

(4) $${Throughput}_{R}=\frac{\left(S_{1}+S_{3}+S_{5}\right)*L_{data}}{\left(T_{e1}-T_{b1}\right)+\left(T_{e3}-T_{b3}\right)+(T_{e5}-T_{b5})}$$

(5) $${Throughput}_{R+}=\frac{\left(S_{2}+S_{4}+S_{6}\right)*L_{data}}{\left(T_{e2}-T_{b2}\right)+\left(T_{e4}-T_{b4}\right)+(T_{e6}-T_{b6})}$$

Among them, $L_{data}$ represents the payload size of each packet, ${Throuthput}_{R}$ represents the average throughput of R within $R_{ol}$, ${Throuthput}_{R+}$ represents the throughput corresponding to $R^{+}$ within $R_{ol}$. If ${Throuthput}_{R}\leq {Throuthput}_{R+}$, it indicates that the oscillation leads to an increase in throughput, and the threshold does not need to be updated to fully utilize the advantages brought by oscillation. If ${Throuthput}_{R}>{Throuthput}_{R+}$, it indicates that the oscillation brings about a decrease in throughput, and it is necessary to reduce the threshold for increasing the rate to half of the original value to impose punishment. This makes the requirements for increasing the rate more stringent to avoid the occurrence of sustained-rate oscillations in the future.

**Figure 3:**
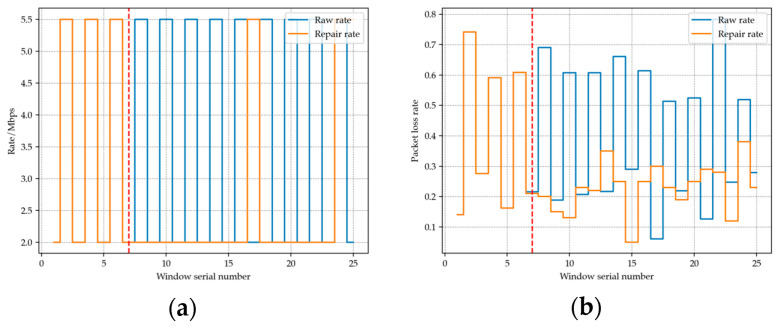
(**a**) Transmission rate comparison before and after adding rate oscillation detection mechanism. (**b**) Packet loss rate comparison before and after adding rate oscillation detection mechanism.

Figure 3 below shows a sequence of rate oscillations that we discovered during the 802.11b simulation process. It can be seen that after the addition of rate oscillation detection mechanism, the fluctuation of the rate has been significantly suppressed and that the packet loss rate has been reduced.

In the first six windows, the rate switches back and forth between 2 Mbps and 5.5 Mbps. After adding the oscillation detection mechanism, in window number 7 (red dotted line), an average throughput evaluation of the oscillation sequence is performed by combining the historical packet length and transmission time. The evaluation results indicate that in this historical sequence, the throughput provided by using the lower rate of 2 Mbps is higher than that provided by 5.5 Mbps. Therefore, a rate of 2 Mbps will be used for transmission in window number 7, and the threshold for increasing the rate will be lowered, making the conditions for increasing the rate more stringent. It can be observed that in the subsequent windows, the rate stabilizes, and the packet loss rate remains low.

## 4. Simulation Results

In this section, we use NS-3 to verify the performance of EAR in different scenarios. First, we designed a series of experiments to validate the necessity of dynamic parameters in the EAR algorithm. Secondly, by constructing a hidden terminal model, we demonstrated the positive impact of the dynamic RTS/CTS mechanism on the performance improvement of the EAR algorithm. Finally, we investigated the influence of node quantity and node movement speed on different algorithms. The final simulation results indicate that the throughput performance of the EAR algorithm is superior to other comparative algorithms in different scenarios.

### 4.1. Case Study—Adjustment of Window and Threshold

To demonstrate the necessity of parameters updates, we designed a set of parameter validation experiments. In this experiment, fixed threshold range and window size were used for throughput testing. This experimental scenario consists of two nodes: an Access Point (AP) node and an STA (Station) node. The UDP traffic of 802.11b 11 Mbps CBR generated by the STA node to the AP node is sent at different distances using the LogDistancePropagationLossModel [29] as the propagation loss model for NS-3. The path loss varies depending on distance, as shown in Equation (6) below.

(6) $$L=L_{0}+10nlog(\frac{d}{d_{0}})$$

where *n* is the path loss distance exponent, $d_{0}$ is the reference distance (m), $L_{0}$ is the path loss at reference distance (dB); and d and L represent the distance and path loss (dB).

**Figure 4:**
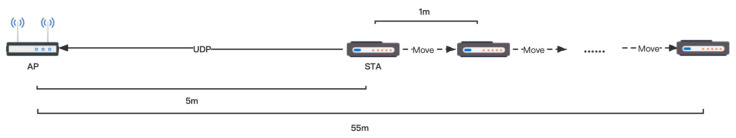
Pull distance scene model.

In the scenario, the movement step of STA is set to 1 m, and the movement time interval is 1 s, which means that the STA node moves away from the AP node at a distance of 1 m per second. STA node starts at the coordinates (5,0,0), and AP node remains at the coordinates (0,0,0) throughout the simulation process. The distance scene model is shown in the following Figure 4.

We repeated the experiment 200 times, and the average throughput under each threshold and window during the entire process is shown in Table 3 below.

It can be seen that the average throughput varies under different threshold ranges and window sizes. When the threshold range is fixed, dynamic windows provide more throughput. When the window size range is fixed, dynamic thresholds also provide more throughput. Finally, when the threshold range and window size can be dynamically adjusted, the throughput provided is the highest. The above experimental results demonstrate that dynamic parameters have better environmental adaptability and provide higher throughput compared to fixed parameters when the channel environment changes significantly.

### 4.2. Case Study—Effect of Hidden Terminal

In order to explore the impact of a hidden terminal on rate selection, this part used the NS-3 platform to build a WLAN environment based on 802.11g. AARF, RBAR, CHARM and EAR algorithms were used for simulation experiments in the hidden terminal environment, and the experimental results were compared and analyzed. During the simulation experiment, to ensure the fairness of the experiment, the sender uses UDP one-way transmission protocol and sends UDP data streams at a CBR (Constant Bit Rate) of 60 Mbps. The specific parameter settings for each module of NS-3 are shown in Table 4.

**Figure 5:**
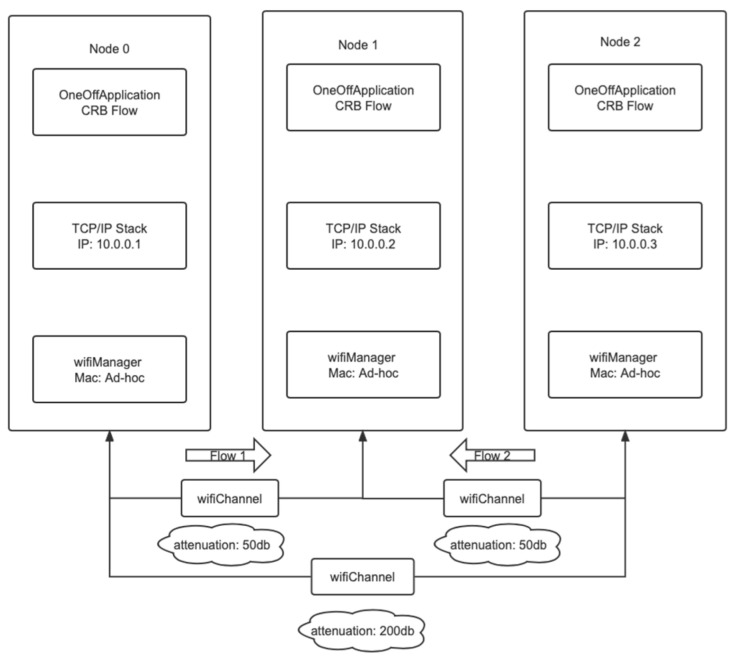
Hidden terminal model.

The model for the hidden terminal model is shown in the following Figure 5.

The channel propagation loss model selection for this experiment is the MatrixPropagationLossModel of NS-3. The propagation loss of each pair of nodes in this model is fixed and does not depend on their actual positions. And by default, the propagation loss is symmetric. When the propagation loss is 50 dB, two nodes can send and receive information to each other. When the propagation loss reaches 200 dB or above, the signals of the two nodes are weak and cannot be detected. This design can effectively construct a hidden terminal experimental model.

The sender uses the OnOffApplication class to create a CBR source, which can generate traffic for a single destination. However, its sending and stopping states are alternated by default. Therefore, setting its OnTime and OffTime properties to 1 and 0, respectively can keep the source in the sending state. The receiving end uses the PacketSink class in NS-3 to install application services for the node, which are responsible for receiving and consuming the traffic generated to the IP address and port. Finally, we install the EarWifiManager algorithm for flow 1 and select ConstantRateWifiManager as the rate algorithm for flow 2.

We conducted 200 simulation experiments, each lasting for 5 s. To facilitate the display of the EAR algorithm operation process, Table 5 below shows a representative set of data.

**Figure 6:**
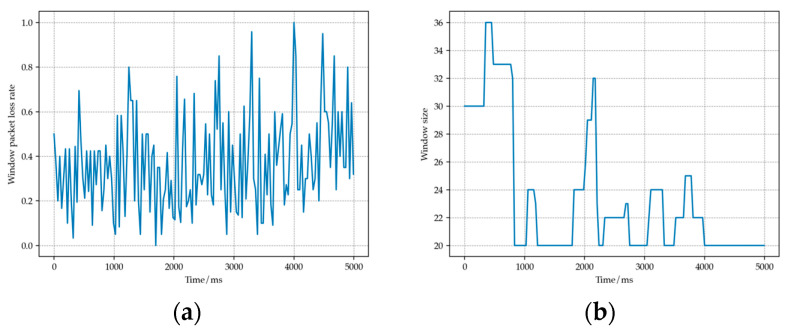

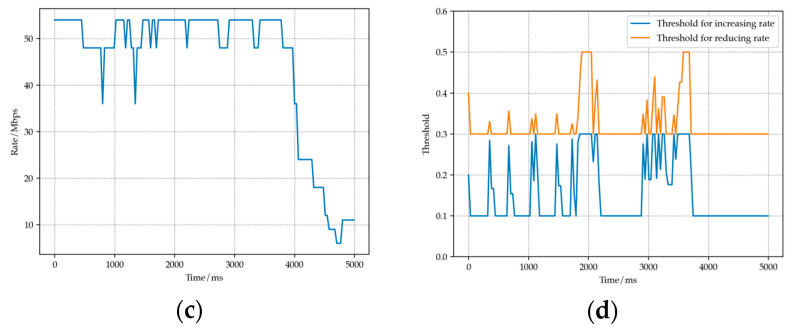
(**a**) Changes in packet loss rate. (**b**) Changes in window size. (**c**) Changes in transmit rate. (**d**) Changes in threshold.

In Flow 1, Node 0 sent a total of 26,785 packets, and 2286 packets were successfully received by Node 1. The average throughput was calculated to be 5.22305 Mbps. In Flow 2, Node 2 sent a total of 26,825 packets, with 1565 successfully reaching Node 1 and an average throughput of 3.57571 Mbps. Meanwhile, this section utilized the tracking system provided by NS-3 to conduct a statistical analysis of the data transmission changes during the Flow 1 experiment, as shown in Figure 6.

**Figure 7:**
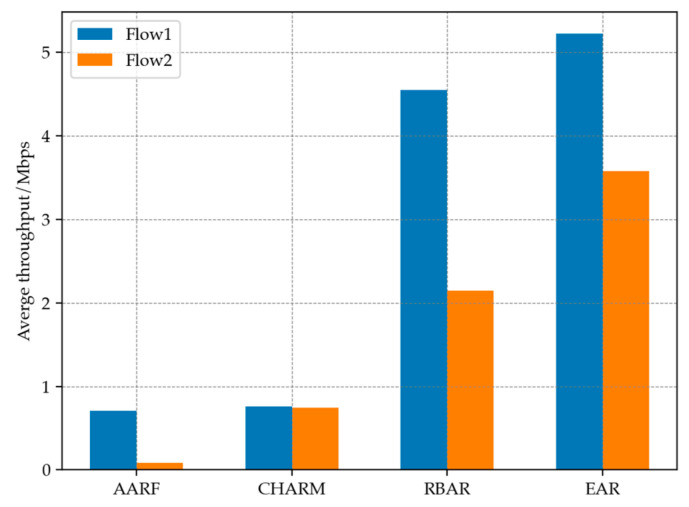
Throughput performance of different algorithms in hidden terminal scenario.

In addition, the model experiment also incorporates AARF, RBAR and CHARM algorithms for simulation, and the comparative performance is shown in the following Figure 7. It can be seen that it is difficult for algorithms that do not have the addition of RTS mechanism to overcome the packet loss problem in the case of a hidden terminal scenario. And the throughput performance of EAR’s adaptive RTS mechanism in this scenario is also better than the RBAR using full RTS mode. The high collision rate caused by hidden terminal can cause AARF and CHARM errors to reduce transmission rates, thereby exacerbating channel degradation. The performance of both on Flow 1 is similar, while on Flow 2, CHARM performs better than AARF. But overall, the throughput provided by both in this scenario is not ideal.

### 4.3. Comprehensive Scenario

**Figure 8:**
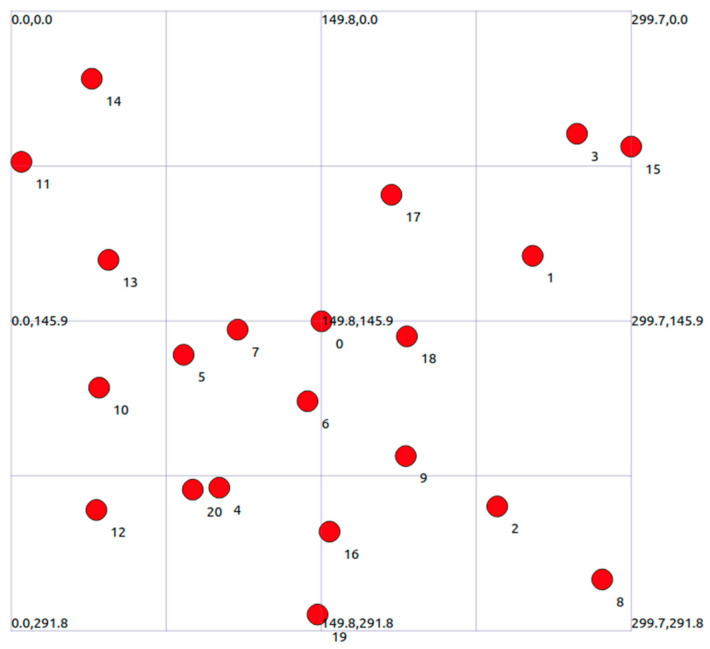
Topological structure of the experimental area.

In the comprehensive testing scenario, we constructed a basic BSS (Basic Service Set) network consisting of one AP and 20 STA. In this network, STA cannot communicate directly with each other and must transmit data through AP. The distribution range of STA and AP is in a rectangular area of 300 m × 300 m. The experimental topology is shown in the following Figure 8.

AP is always at a fixed position in the center of the region (150, 150, 0), and STA is randomly distributed within the region. Each STA continuously generates a 100 Mbps CBR data stream. At the same time, in order to be closer to the real scene, the LogDistancePropagationLossModel is introduced in the environment to perform signal fading on STA nodes at different distances.

#### 4.3.1. Different STA Quantities

**Figure 9:**
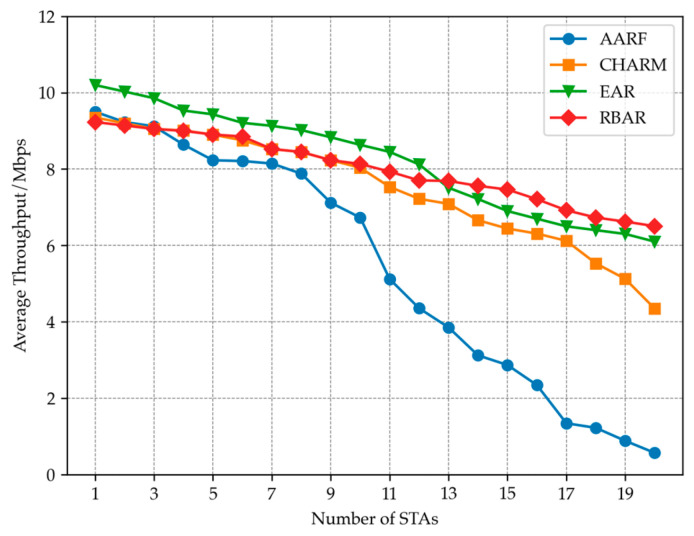
Adding nodes in the WLAN, the average throughput changes of different algorithms.

This group of experiments verified the impact of different STA numbers on WLAN system throughput by setting different numbers of STA access AP in the above areas. Figure 9 shows the performance of four rate adaptive algorithms, EAR, AARF, CHARM and RBAR, as the number of STA nodes increases. It can be seen that as the number of STA sites increases, the average throughput shows a downward trend. This is because as the number of STA increases, the likelihood of collisions between STAs also increases. At the same time, the average time for each STA to access the AP will also decrease, which will lead to a decrease in system average throughput. Among them, the AARF algorithm is the most sensitive to changes in the number of STAs, and its throughput decreases the fastest. The EAR algorithm, due to the addition of the RTS adaptive mechanism, exhibits relatively good stability and maintains relatively high throughput even when the number of STAs is high. Overall, the RBAR algorithm exhibits the most stable throughput performance during the process of node increase, and when the number of nodes exceeds 13, the average throughput exceeds that of the EAR algorithm. This is due to the RBAR algorithm using RTS for channel reservation before each packet is sent. As the number of STAs in WLAN continues to increase, the probability of packet collisions increases, and the benefits brought by RTS become more apparent. When the number of STAs is less than 10, the throughput performance of CHARM and RBAR is extremely similar. However, as the number of STAs increases, the fading trend of CHARM is more pronounced than that of EAR and RBAR due to the lack of collision detection mechanism.

#### 4.3.2. Different Mobile States

**Figure 10:**
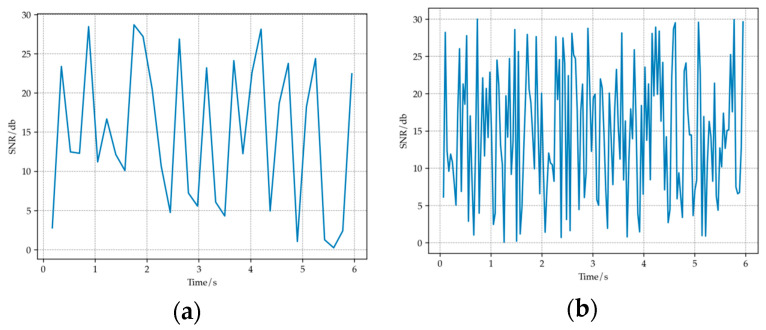
(**a**) Changes in SNR over time when the movement speed is slow. (**b**) Changes in SNR over time when the movement speed is fast.

The movement of nodes can cause multipath effects, leading to changes in the channel environment [30]. Figure 10 shows the SNR curves corresponding to two different node movement speeds in the simulation experiment. It can be seen that as the speed of node movement increases, the fluctuation of SNR becomes more severe, channel fading also becomes more severe, and the overall channel environment becomes worse. This is because the movement of nodes can cause changes in frequency and phase, leading to signals traveling through different channel propagation paths. Due to different path lengths and scattering characteristics, signal synthesis or interference may occur, thus affecting the effectiveness of the channel. When the node moves faster, the time interval between adjacent signal transmissions is shorter, and the overlap and interference between signals become more significant, thereby more severely affecting channel quality.

By configuring the RandomWalk2dMobility model of NS-3, STA nodes were randomly moved within the aforementioned 300 × 300 m area and simulated two different motion states: When the motion speed is 0.5 m/s, the state of slow node movement is simulated; and when the motion speed is 5 m/s, the state of rapid node movement is simulated.

**Figure 11:**
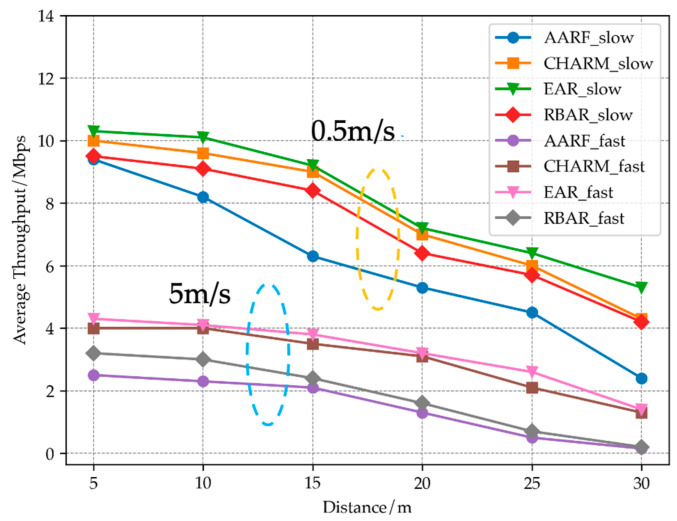
Comparison of throughput of four algorithms at different speeds and distances.

Figure 11 shows the average throughput variation of STA at different speeds and distances. It can be seen that when the STA mobility speed is slow, the EAR algorithm has higher throughput than AARF, RBAR and CHARM algorithms at different distances, and as the distance increases, the advantage is most obvious when reaching a distance of 30 m. This is because when the channel state of STA is relatively stable, the EAR algorithm tends to adopt a high transmission rate and maintain a relatively stable rate. The dynamic window and threshold update strategy ensure that the EAR algorithm can accurately capture changes in the channel. In high-speed mobile mode, the throughput of all four algorithms decreases sharply, with an average throughput only reaching about half of that at low speeds. At 10 m, 20 m and 30 m, the throughput performance of EAR algorithm and CHARM algorithm is similar thanks to CHARM’s channel evaluation and rate adaptation mechanism. AARF and RBAR perform poorly in this scenario, only reaching about half of the throughput of EAR algorithm.

Based on mobile scenarios, compared to the CHARM, RBAR and AARF algorithms, EAR has increased UDP’s throughput by 7%, 25% and 51%, respectively, improving the throughput performance of wireless networks in mobile environments.

## 5. Conclusions

The proposed EAR rate adaptation algorithm, as outlined in this article, effectively identifies the source of channel degradation and differentiates whether the degradation is due to hidden terminal or low SNR. Diverse rate adjustment measures can be implemented based on the various channel environments. The dynamic window and threshold adjustment mechanism significantly enhance the real-time and accuracy of rate adjustment compared to certain conventional rate adaptive algorithms that are currently available. However, there are still shortcomings in the current work. The design concept of EAR is based on the characteristics of traditional standards, such as 802.11a/b/g. There has been no targeted adaptation and optimization of relevant standards, such as 802.11n/ac/ax. This is also a common limitation of many rate adaptation algorithms at present. In future work, EAR will make corresponding improvements based on the characteristics of different standards. For example, the 802.11n standard has added new features, such as aggregate frames, MIMO (Multiple Input Multiple Output), channel bonding and ShortGI (Short Guard Interval). Additionally, 802.11 n allows for many more rate options than 802.11a/b/g mode, ranging from 6.5 Mbps to 600 Mbps. As the rate increases, loss does not monotonically grow with rates in different modes. Therefore, future work has to take these additional factors into account. In addition, 802.11e enhances its support for QoS (Quality of Service) by setting priorities, which can effectively ensure the quality of service for different priority services. In 802.11e devices, the competition unit of the channel in EDCA (Enhanced Distributed Channel Access) mode changes from a set of data to a transmission opportunity. Therefore, the unit of the statistical window in EAR also changes from the number of data packets to a period of time. Additionally, threshold initialization and threshold update strategies are implemented under four different AC (Access Category) conditions to meet the requirements for QoS.

## Figures and Tables

**Table 1:** Classic algorithm classification.

Algorithm	Sender or Receiver	History or Current
ARF [12]	Sender	History
AARF [13]	Sender	History
Sample Rate [14]	Sender	History
RBAR [15]	Receiver	Current
OAR [16]	Receiver	Current
CHARM [17]	Sender	Current
CARA [18]	Sender	History
SGRA [19]	Sender	Current
BARA [20]	Sender	Current
Minstrel [21]	Sender	History

**Table 2:** Initialization parameters.

Parameter	Symbol	Value
Initial rate	$R_{org}$	Protocol maximum rate
Initial window size	${Wind}_{org}$	30
Maximum window size	${Wind}_{max}$	40
Minimum window size	${Wind}_{min}$	20
Original decrease rate threshold	$P_{down\_org}$	0.4
Original increase rate threshold	$P_{up\_org}$	0.2
Threshold update coefficient	α	0.2

**Table 3:** Comparison of throughput between fixed and dynamic parameters in high dynamic channel environments.

	Window	20	30	40	Dynamic
Threshold					
0.1–0.2		2.33 Mbps	2.43 Mbps	2.12 Mbps	2.54 Mbps
0.1–0.3		2.54 Mbps	2.68 Mbps	2.55 Mbps	2.74 Mbps
0.1–0.4		2.43 Mbps	2.83 Mbps	2.63 Mbps	3.12 Mbps
0.2–0.3		2.21 Mbps	2.44 Mbps	2.36 Mbps	3.01 Mbps
0.2–0.4		2.12 Mbps	2.88 Mbps	2.34 Mbps	2.97 Mbps
0.3–0.4		1.74 Mbps	2.13 Mbps	2.29 Mbps	2.53 Mbps
Dynamic		2.64 Mbps	3.26 Mbps	3.03 Mbps	3.45 Mbps

**Table 4:** Parameter settings for each module of NS-3.

Parameter	Value
Protocol standard	IEEE 802.11g
Frequency	2.4 GHz
Physical layer model	YansWifiPhy
Transmission method	CBR
Data flow type	UDP
Packet length	1400 Bytes
Data flow rate	60 Mbps
Channel model	YansWifiChannel
Channel fading model	MatrixPropagationLossModel

**Table 5:** Performance of the EAR on each data stream.

Indicators	Flow 1	Flow 2
Tx Packets	26,785	26,825
Tx Bytes	38,248,980	38,306,100
Tx Offered	61.1984 Mbps	61.2898 Mbps
Rx Packets	2286	1565
Rx Bytes	3,264,408	2,234,820
Average Throughput	5.22305 Mbps	3.57571 Mbps

## Data Availability

Not applicable.
